# Targeted elimination of mesenchymal-like cancer cells through cyclic stretch activation of Piezo1 channels: the physical aspects

**DOI:** 10.1007/s12551-025-01304-y

**Published:** 2025-03-19

**Authors:** Ivana Pajic-Lijakovic, Milan Milivojevic, Boris Martinac, Peter V. E. McClintock

**Affiliations:** 1 Department of Chemical Engineering, Faculty of Technology and Metallurgy, University of Belgrade, Belgrade, Serbia; 2 Mechanosensory Biophysics Laboratory, Victor Chang Cardiac Research Institute, Sydney, Australia; 3 Faculty of Medicine, St Vincent’S Clinical School, University of New South Wales, Sydney, NSW Australia; 4 Department of Physics, Lancaster University, Lancaster, LA1 4YB UK

**Keywords:** Bilayer bending, Membrane viscoelasticity, Bilayer-cytoskeleton coupling, Mechanosensitive Piezo1 channels, Intracellular calcium oscillations

## Abstract

The application of cyclic stretch could represent a novel therapeutic method for fighting cancer. Research indicates that this mechanical stimulus selectively induces cell death in cancer mesenchymal-like cells while enhancing the migration and proliferation of healthy epithelial cells. Although the mechanisms have been examined through the lenses of cell signalling, gene expression, and biochemical processes, a significant gap persists in our understanding of the physical factors that drive cellular responses. This study aims to clarify the importance of physical factors, particularly the viscoelastic characteristics of the cell membrane, including actin cytoskeleton and lipid bilayer, and how their coupling affects bilayer bending and activation of the mechanosensitive Piezo1 channels in response to cyclic stretch in both epithelial and cancer cells. The bending of the bilayer surrounding Piezo1 molecules affects their conformations, which in turn influences calcium influx. This bending is contingent upon the coupling between the cell membrane and extracellular matrix. The primary factors contributing to the mechanically induced apoptosis of cancer cells are the perturbation of intracellular calcium homeostasis and disruption of focal adhesions.

## Introduction

The efficiency of various therapies against cancer diseases such as radiation and chemotherapies is usually limited. The main disadvantages of these therapies are (i) nonselective damage of epithelial and cancer cells and (ii) enhanced transition from proliferative to quiescent state of cancer cells. Quiescent cancer cells, which are defined as ‘dormant’, non-dividing, and inactive within the cell cycle, are considered a critical factor in the ineffectiveness of cancer pharmacotherapies (Lindell et al. 2023). Ionizing radiation may facilitate the migration and invasion of cancer cells through complex interactions within the microenvironment, including alterations in cell–cell junctions, modifications to extracellular matrix connections, secretion of proteases, and the induction of epithelial–mesenchymal transition (Moncharmont et al. 2014; Lacombe and Zenhausern 2022). However, mechanical stimulation caused by cyclic stretch protects healthy tissue against irradiation-induced damage of DNA to some extent. Nagayama and Fukuei (2020) demonstrated that a cyclic stretch applied at a frequency of 0.5 Hz and an amplitude of 10% over a duration of 10 h provided protection to NIH3T3 fibroblasts from nuclear DNA damage induced by ultraviolet radiation.

Cyclic mechanical loads, characterized by stretching or compression within the frequency range of ${10}^{-2}-{10}^{2}$ Hz and amplitudes reaching up to 30%, have been demonstrated to selectively trigger apoptosis in cancer cells. Concurrently, these loads facilitate the migration and proliferation of epithelial cells over durations spanning several minutes to a few hours (Olcum and Ozcivici 2014; Takao et al. 2019; Spencer et al. 2021; Tijore et al. 2021; 2022). Notably, the experiments did not detect any quiescent cancer cells. The majority of these studies have focused on breast epithelial and cancer cells, conducted under in vitro conditions utilizing various substrate matrices. The effectiveness of the cyclic mechanical load is influenced by the stiffness of these extracellular matrices (Bavi et al. 2019; Tijore et al. 2021; Yang et al. 2020). Tijore et al. (2021) pointed out that cyclic stretching reduces proliferation of MDA-MB-231 cancer cells more efficiently on a stiffer matrix than on a soft one. Static mechanical stress, akin to slowly varying stress, contrasts with cyclic mechanical load by promoting the migration and proliferation of cancer cells while inhibiting those processes in epithelial cells. For instance, a compressive stress of 773 Pa has been shown to hinder the movement of breast cancer cell lines MCF-10A and MCF-7, while simultaneously enhancing the motility of more aggressive cell lines such as 4T1 and MDA-MB-231, as well as 67NR cells (Tse et al. 2012). Furthermore, the accumulation of solid mechanical stress within the central region of co-cultured epithelial-cancer spheroids encourages the migration of cancer cells from the core towards the surface region, while concurrently reducing the migration of epithelial cells (Devanny et al. 2021; Pajic-Lijakovic et al. 2024a). Riehl et al. (2020) investigated and contrasted the responses of MDA-MB-231 and MDA-MB-468 breast cancer cell lines alongside MCF-10A epithelial cells when subjected to a low shear stress of 1.5 Pa. The application of shear stress was found to promote the directional movement of MDA-MB-231 cells, whereas it inhibited the mobility of MCF-10A epithelial cells. Additionally, uniaxial stretching, resulting from the directional collective migration of epithelial monolayers, leads to the accumulation of residual tensile stress and an increase in the surface energy of these monolayers, which in turn limits further extension (Pajic-Lijakovic et al. 2024b). The surface energy is influenced by the tension of the epithelial surface and the expansion of the monolayer surface area (Pajic-Lijakovic et al. 2023). In contrast, cancer cells exhibit markedly different behaviours. To gain a more profound understanding of the differential responses exhibited by epithelial and cancer (mesenchymal) cells when subjected to internally generated mechanical stress during collective cell migration, it is essential to examine the viscoelastic properties of both cell types at a supracellular level. Epithelial cells form strong E-cadherin-mediated cell–cell adhesion, resulting in the migration of tightly connected cell clusters. The rheological behaviour of migrating epithelial collectives corresponds to viscoelastic solids and change with an increase in epithelial packing density (Pajic-Lijakovic et al. 2024b). In contrast to epithelial cells, cancer (mesenchymal) cells establish weak cell–cell adhesion contacts and migrate as streams. It is in accordance with the fact that epithelial cells establish strong E-cadherin-mediated cell–cell adhesion contacts, while mesenchymal cells establish weak N-cadherin-mediated cell–cell adhesion contacts (Barriga and Mayor 2019). Their migration is more dissipative and corresponds to viscoelastic liquids (Pajic-Lijakovic 2021). The surface tension of cancer cells is much lower than the surface tension of epithelial cells (Devanny et al. 2021; Pajic-Lijakovic et al. 2023). As a result, two physical factors, namely (i) dissipative cell migration and (ii) low surface tension, promote the movement of cancer cells on substrate matrices. The influence of these physical factors is contingent upon the strength of cell–cell and cell–matrix adhesion contacts, as well as the contractility of the cells (Devanny et al. 2021; Pajic-Lijakovic et al. 2024b). Furthermore, these dynamics are influenced by the functionality of mechanosensitive Piezo1 channels, which influence remodelling of adhesion contacts (Tijore et al. 2021).

To achieve a deeper insight into the sensitivity of cancer cells to cyclic stretching, it is imperative to analyze the cause-consequence relationship between the viscoelastic characteristics of the cell membranes in epithelial and cancer cells and the activity of Piezo1 channels (Martinac, et al. 2020). Piezo1 channels are pivotal in managing the strength of adhesion between cells and the extracellular matrix, as well as in controlling intracellular calcium levels, which are essential for various cellular processes, including migration, proliferation, and division (Tijore et al. 2021; Singh et al. 2021; Ridone et al. 2019a, b). The primary objective of this theoretical analysis is to highlight the influence of the viscoelastic properties of the actin cytoskeleton, a poorly characterized part of the actin cytoskeleton seemingly playing key roles in cell mechanics, on the bending of the lipid bilayer in the vicinity of Piezo1 channels. This interaction subsequently affects the activity of these channels and the associated calcium influx. To investigate the cyclic stretch activation of Piezo1 channels, it is essential to take into account several factors: (i) the influence of membrane viscoelasticity on the rearrangement and activation of Piezo1 channels; (ii) the coupling between the extracellular matrix, the lipid bilayer, and the cytoskeleton in relation to membrane viscoelasticity; (iii) the effects of different frequencies on the viscoelastic properties of the membrane by determining the role of cholesterol; and (iv) the role of Piezo1 channel activation in the intercellular oscillation of calcium ions as one of the main factors that govern cell response under cyclic stretching.

## Mechanical cyclic stretch of epithelial and cancer monolayers

It has been documented in various experimental systems that cyclic mechanical stretching selectively induces cell death in cancer cells, while enhancing the migration and proliferation of epithelial cells (Spencer et al. 2021; Tijore et al. 2021; 2022). Frequencies within the range of ${10}^{-2}-{10}^{3}$ Hz, corresponding to time scales from minutes to milliseconds, have been utilized, with amplitudes of up to 30% and exposure times varying from several tens of minutes to several hours. Furthermore, the response of cells to cyclic stretch is contingent upon the stiffness of the substrate matrix (Zhang et al. 2006; Tijore et al. 2021).

Various frequencies and their corresponding temporal scales excite specific elements of the cellular structure, as shown in Table 1.

**Table 1:** Frequencies of stretching and corresponding strains are presented together with oscillation frequencies of different cellular structures

Characteristic oscillation frequencies of different cellular structures	Frequencies of stretching	Strain	Cell type	Cell response	Reference
Inter-filament interactions (Pajic-Lijakovic and Milivojevic 2015; Pritchard et al. 2014) Gating of ion channels (Lewis et al. 2017)	${10}^{3}-{10}^{5}$Hz	(-)	MDA-MB-231		Singh et al. (2021)
Intra-filament interactions (Heinrick and Sackmann 2006) Change of the bending state of the bilayer (Braunmuuller et al. 2012) Coupling between the cytoskeleton and bilayer (Pajic-Lijakovic and Milivojevic 2015)	$1-{10}^{3}$Hz	7.5% and 15%	HUVECs MDA-MB-231 MCF-7		Spencer et al. (2021)
		7.2–27%	MDA-MB-231		Olcum and Ozcivici (2014)
Shape oscillations of passive (resting cells) (Thoumine and Ott 1997 )	Hz	10%	Caco-2 IEC-6	$\uparrow$ (on collagen I matrix)	Zhang et al. (2006)
				$\uparrow$ (on collagen I matrix)	
Rearrangement of cytoskeleton domains (Pajic-Lijakovic and Milivojevic 2014)		5%	MDA-MB-231	$\downarrow$	Tijore et al. (2021)
			SKOV3	$\downarrow$	
			HT1080	$\downarrow$	
			HEK293T	$\downarrow$	
			MCF10A	$\uparrow$	
		10%	MDCK	$\downarrow$	Dow et al. (2024)
			T151	$\sim$	
		10%	HCT116	$\uparrow$	Strelez et al. (2021)
			HT29	$\sim$	
Actomyosin contractions (Gorfinkiel and Blanchard 2011) Cadherin turnover (Lee and Wolgemuth 2011) Intracellular oscillations of calcium (Liang et al. 2022a, b) Phosphorylation and glycosylation of membrane proteins (Petrungaro et al. 2019)	$\sim {10}^{-2}$Hz	1.4%	MDA-MB-231	$\downarrow$	Yadav et al. (2019)

In particular, a time scale on the order of minutes is associated with cellular contractions, while a time scale measured in seconds relates to inter-filament interactions and the broader lateral diffusion of transmembrane proteins and lipids (Pajic-Lijakovic and Milivojevic 2015). The frequency of actomyosin contractions is 0.33–0.5 cycles/min (Gorfinkiel and Blanchard 2011). The application of cyclic stretch at a frequency of ${10}^{-2}$ Hz, which aligns with the internal frequency of cellular contractions, can produce resonant effects that enhance the amplitude of oscillations. Interestingly, cyclic stretch at a very low frequency of ${10}^{-2}$ Hz and amplitude of 1.4% within 4 h also results in apoptosis of cancer MDA-MB-231 cells primarily caused by disruption of FAs (Yadav et al. 2019). Dow et al. (2024) highlighted the significance of vinculin in enhancing the adhesion between cells and the extracellular matrix during cyclic stretching. Additionally, intra-filament interactions and the lateral movement of transmembrane proteins and lipids within mesoscopic regions, as well as gating of ion channels, occur over a time scale from nanoseconds to milliseconds. Singh et al. (2021) revealed that low-frequency ultrasound of 33 kHz caused calcium entry through mechanosensitive Piezo1 channels that disrupt microtubules via calpain protease activation in cancer cells, while epithelial cells retained their intact state. The resistance exhibited by epithelial cells may be associated with the rigidity of the epithelial cytoskeleton, which is notably greater than that observed in cancer cells (Tijore et al. 2021).

Cyclic stretching of epithelial MCF10A and cancerous MDA-MB-231 monolayers at a frequency of 0.5 Hz and a strain of 5% over a duration of 6 h on a rigid substrate led to (i) a 2.5-fold reduction in the proliferation rate of MDA-MB-231 cells and (ii) an enhancement in the proliferation rate of epithelial MCF10A cells (Tijore et al. 2021). A similar outcome was observed at a frequency of 1 Hz (Tijore et al. 2021). SKOV3 cells (human ovarian adenocarcinoma), HT1080 cells (human fibrosarcoma), and HEK293T cells (transformed human embryonic kidney cell) behave similarly as breast MDA-MB-231 cancer cells under the same conditions (Tijore et al. 2021). In contrast to widely explored MDA-MB-231 cells, highly aggressive HCT116 colorectal cancer cell invasion is enhanced by cyclic stretching at a frequency of 0.2 Hz and a strain of 10%, while less invasive HT29 is unaffected (Strelez et al. 2021). Additionally, Spencer et al. (2021) demonstrated that cyclic stretching at a frequency of 1 Hz and an amplitude of 7.5% resulted in a decreased expression of genes linked to proliferation when compared to MDA-MB-231 cells cultured under static conditions. Cyclic stretching at a frequency of 0.3 Hz and an amplitude ranging from 7.2 to 27% promotes the closure of wounds in epithelial Caco-2 and IEC-6 cells when cultured on a fibronectin substrate, while it exhibits a modest reduction in wound closure on a collagen substrate (Zhang et al. 2006). The effectiveness of cyclic stretching may therefore provide a supplementary therapeutic approach in the treatment of cancer.

Cyclic stretching of MDA-MB-231 cells at a frequency of 90 Hz for 15 min/day, 5 days a week, under ambient conditions leads to cell cycle arrest rather than inducing apoptosis (Olcum and Ozcivici 2014). This frequency exceeds the characteristic frequency for the gel-to-sol transition of the MDA-MB-231 cell membrane, which is approximately ${\text{f}}_{\text{c}}\sim 20\text{ Hz}$ (Rother et al. 2014). Following the initial week of culture, the cyclic stretch significantly decreased both the number and viability of the cancerous MDA-MB-231 cells, while the non-cancerous MCF10A cells remained unaffected (Olcum and Ozcivici 2014). The implications of these findings are examined with a focus on the function of tropomyosin 2.1-based contractile units (CUs) (Tijore et al. 2021).

The contractile units (CUs) represent a cell rigidity-sensing complex, characterized as a modular structure measuring 2–3 μm in size. These units assemble at the periphery of the cell upon initial contact with a substrate matrix, prior to the establishment of stress fibres or other subsequent cytoskeletal structures (Yang et al. 2020). Composed of myosin IIA, actin filaments, tropomyosin 2.1 (Tpm 2.1), α-actinin 4, and various other cytoskeletal proteins, these contractile units play a crucial role in modulating the strength of cell–matrix focal adhesions in relation to the stiffness of the substrate. On rigid substrates, CUs facilitate the formation of mature focal adhesions (FAs), which are often associated with increased cellular proliferation (Yang et al. 2020). Conversely, on softer substrates, the contractions exhibited by these units are very short-lived, resulting in a rapid disassembly of adhesions and ultimately leading to cell death through the mechanism of anoikis, which refers to cell death induced by the disruption of focal adhesions and subsequent cell detachment.

Yang et al. (2020) and Tijore et al. (2021) identified a significant relationship between the diminished ability to sense rigidity and the process of cellular transformation to cancer phenotype. Epithelial MCF10A cells are capable of forming cellular units (CUs), whereas MDA-MB-231 cells are deficient in the cytoskeletal protein tropomyosin 2.1, which is essential for effective rigidity sensing (Tijore et al. 2021). Human foreskin fibroblasts (HFF) exhibited the formation of 39 CUs per 100 μm^2^ on rigid pillars (*k* = 8.4 pN nm^−1^) and 24 CUs per 100 μm^2^ on soft pillars (*k* = 1.6 pN nm^−1^) within a 10-min period during initial spreading. In contrast, all cancer cell lines generated fewer than 2 CUs per 100 μm^2^ within the same timeframe on both types of pillars (Yang et al. 2020).

Reduced number of CUs in cancer cells causes formation of smaller FAs in comparison with epithelial cells leading to more intensive cell tractions (Tijore et al 2021). These increased traction forces can stimulate mechanosensitive Piezo1 channels, thereby perturbing calcium influx. The application of cyclic stretch promotes Piezo1-mediated calcium entry, which subsequently triggers calpain2-driven mitochondrial apoptosis in MDA-MB-231 cells (Tijore et al. 2021). The process of cellular apoptosis is facilitated by the accumulation of calcium ions within the cells and the disruption of intracellular calcium compartmentalization (Kim and Hyun 2023). It has been shown that Piezo1 expression is higher in most cancer cells compared to normal cells leading to higher sensitivity of cancer to mechanical stress (Kim and Hyun 2023; Yu and Liao 2021). Furthermore, Piezo1 channels are highly expressed in various mammalian cells and tissues, including lungs, blood vessels, smooth muscle, and bladder, for example, with a preference for the endothelium (Qin et al. 2021). It is crucial to investigate the effects of both inter- and intracellular calcium oscillations on various cellular functions, particularly by emphasizing the differences between epithelial and cancerous cells. This knowledge is essential for understanding how cells respond to cyclic stretching.

## Inter- and intracellular calcium oscillations

The regulation of intracellular calcium concentration is essential for a variety of cellular functions, including proliferation and migration. The process of cell migration relies on a calcium gradient that is established from the rear to the front of the cell, while low oscillations in cytosolic calcium levels are critical for the formation of invadopodia. Additionally, sustained increases in cytosolic calcium or excessive mitochondrial calcium can activate necroptotic or apoptotic pathways (Pratt et al. 2018). Furthermore, cells create a supracellular gradient of intracellular calcium concentration between the cells at the wound edge and their neighbouring cells. This gradient results from the complex interactions between intracellular Ca^2+^ signalling and the distribution of calcium within the cells. Lee and Hong (2021) highlighted the role of intracellular Ca^2+^ signalling in modulating cellular movement through various proteins that facilitate migration, such as myosin light chain kinase, myosin II, calpain, Ca^2+^/calmodulin-dependent protein kinase II, and focal adhesion kinase. In breast cancer cells, the influx of Ca^2+^ induced by Piezo1 activates the Akt/mTOR signalling pathway, which is crucial for the regulation of cell motility and survival (Dombroski et al. 2021). The precise coordination of the assembly and disassembly of integrin-mediated focal adhesions (FAs) is vital for effective cell migration, with the disassembly process being dependent on Ca^2+^ influx (D’Souza et al. 2020). Yao et al. (2022) highlighted the significance of mechanosensitive Piezo1 channels in the regulation of focal adhesions (FAs). The influx of Ca^2+^ not only affects the strength of FAs but also influences the integrity of cell–cell adherens junctions (AJs). These junctions consist of cadherins associated with proteins such as p120 and α-, β-, and γ-catenin. Ninomiya et al. (2024) noted that Ca^2+^ influx is crucial for maintaining the integrity of the actin cytoskeleton associated with AJs. The remodelling of actin induced by mechanical forces is essential for the dynamics and restructuring of cell–cell junctions. The cytoplasmic concentration of Ca^2+^ is typically regulated to approximately 100 nM, which is significantly lower—by a factor of 20,000 to 100,000—than the concentrations found in the extracellular environment (Clapham 2007).

Epithelial cells and cancer cells exhibit markedly distinct behaviour concerning the regulation of intracellular calcium concentrations. Epithelial cells possess the capability to maintain their intracellular calcium levels within an optimal range under physiological conditions. In contrast, cancer cells, including metastatic human prostate and breast cancer cells such as PC-3M and MDA-MB-231, experience spontaneous fluctuations in their intracellular calcium concentrations (Rizaner et al. 2016). The frequency of intracellular Ca^2+^ oscillations corresponds to $\sim 0.01\text{ Hz}$ (Liang et al. 2022a, b). Ion channels located on the cell membrane, including transient receptor potential (TRP) channels, store-operated calcium channels (SOCCs), Piezo 1 mechanosensitive ion channels, inositol-(1,4,5)-trisphosphate (IP3) receptors (IP3Rs), and ryanodine receptors (RyRs), play a crucial role in the regulation of calcium ion concentrations (Velasco-Estevez et al. 2020; Lee and Hong 2021; Mound et al. 2017). The internal dynamics of these ion channels are modulated by ATP, while external factors such as mechanical stimuli also influence their activity. The activation of IP3R1 leads to the generation of rapidly damped calcium oscillations, whereas stimulation of IP3R2 results in consistent and pronounced calcium oscillations. In contrast, IP3R3 acts as a suppressor of calcium oscillations (Mound et al. 2017). Additionally, Rizaner et al. (2016) examined the contribution of voltage-gated sodium channel activity to the initiation of spontaneous intracellular calcium oscillations.

Mechanical strain can perturb intercellular Ca^2+^ concentration. Sauer et al. (2000) reported that mechanically induced intracellular Ca^2+^ waves spread through prostate cancer cells with the velocity of $15\;\mu m/s$. Wave propagation occurs independently of factors such as membrane potential, extracellular calcium ion concentration, the microtubular network, or the presence of functional gap junctions (Sauer et al. 2000). The mechanism involved is associated with the release of ATP, which in turn activates ion channels. In addition to the purinergic P2 receptors mentioned by Sauer et al. (2000), Piezo 1 ion channels may also play a role. It is well established that calcium signalling in cancer cells is influenced by a variety of mechanical stimuli, including cyclic stretch, localized membrane traction, fluid shear, and compression (Tijore et al. 2021; Liang et al. 2022a, b). The influx of calcium ions through Piezo1 channels has been identified as a trigger for calpain 2-dependent apoptosis via the BAX protein, leading to the mitochondrial activation of caspase 3, which is a key mechanism in the cancer response (Tijore et al. 2021). Furthermore, Liang et al. (2022a, b) highlighted the roles of calcium ions, yes-associated protein (YAP), and microRNAs (miRNAs) as significant factors in the response of cancer cells to mechanical loading, whether induced internally or externally.

The mechanosensitive Piezo1 channels have emerged as a primary factor in responses of mammalian cells to mechanical stress or strain (Coste et al. 2012; Gottlieb and Sachs 2012; Bae et al. 2013; Zeng et al. 2018; Kefauver et al. 2020). Thus, it is essential to pinpoint the critical elements that lead to the activation of Piezo1 channels, as these factors elucidate the varying responses seen in cancerous cells compared to epithelial cells.

## Rearrangement and activation of Piezo1 ion channels

Cyclic mechanical stretching of multicellular systems has been shown to activate Piezo1 ion channels (Tijore et al. 2021; Singh et al. 2021). The mechanical forces responsible for the activation of Piezo1 are derived from the phosphorylation of myosin II by myosin light chain kinase (Ellefsen et al. 2019) and by externally applied force. These Piezo1 ion channels serve as frequency filters for cyclic mechanical stimuli, resulting in a progressive reduction in the amplitude of oscillating Ca^2+^ influx with each successive cycle (Lewis et al. 2017). Notably, the difference in current amplitude between the first and second cycle increases significantly with frequency. Research by Lewis et al. (2017) indicates that Piezo1 is not an effective transducer for low-frequency stimuli (< 2 Hz), yet it demonstrates high efficiency in damping high-frequency stimuli (> 20 Hz).

Oscillatory mechanical stress or strain has been shown to promote the proliferation and migration of healthy epithelial cells, while simultaneously inducing calcium-dependent apoptosis, referred to as mechanoptosis, in cancer cells (Tijore et al. 2021). Notably, cyclic stretch enhances the expression of Piezo1 in cancer cells but does not have the same effect on epithelial cells (Tijore et al. 2021). The substantial influx of Ca^2+^ through mechanically activated Piezo1 channels leads to the disruption of microtubules and the activation of a calpain-dependent mitochondrial pathway (Singh et al. 2021). The analysis in this study aims to provide a deeper understanding of the rearrangement of Piezo1 and to elucidate the distinctions between epithelial and cancer cells.

Piezo1 molecules are large, homotrimeric membrane proteins, which comprise 2547 amino acids per monomer (Guo and MacKinnon 2017; Saotome et al. 2018; Dombroski et al. 2021; Yang et al. 2022). The channel complex consists of three propeller-like blades on the extracellular region of the membrane, a single extracellular cap, an intracellular anchor region, three intracellular beam regions, and finally, a pore conducting path (Ridone et al. 2019a, b; Dombroski et al. 2021). Piezo1 exhibits a structural configuration resembling a triskelion, measuring 24 nm in diameter and 9 nm in depth, which results in an estimated total projected area of around 450 nm^2^ (Mulhall et al. 2023). These molecules migrate laterally over the cell membrane. The population of Piezo1 located near the FAs can be activated by cell tractions (Ellefsen et al. 2019). Vaisey et al. (2022) considered lateral migration of Piezo1 over the membrane of erythrocyte and pointed out that Piezo1 molecules (i) do not form clusters with itself; (ii) cannot attach to F-actin, Spectrin, and the Gardos channel; and (iii) are in-homogeneously distributed over the membrane. The average number of Piezo1 molecules per single erythrocyte is $\sim 80$, while the number of transmembrane protein Band 3 is much larger and equal to $\sim \text{1,000,000}$ (Pajic-Lijakovic and Milivojevic 2015; Vaisey et al. 2022). Piezo1 molecules exhibit Brownian diffusion within the actin cytoskeleton micro-domains and hop diffusion between two domains on a time scale of milliseconds (Vaisey et al. 2022). Successive hops induce damping effects in the migration of Piezo1 molecules (Vaisey et al. 2022). Long-time diffusion accounts for cumulative effects of successive hops and corresponds to sub-diffusion. The sub-diffusion occurring on a time scale of seconds can be expressed as:

1 $${D}_{t}^{{a}_{p}}{c}_{\text{p}}\left(t\right)={D}_{\text{eff}}{\nabla }^{2}{c}_{\text{p}}$$

where ${c}_{\text{p}}\left(t\right)$ is the surface number density of Piezo1 molecules, ${D}_{\text{eff}}$ is the effective diffusion coefficient, ${D}_{t}^{a} \left(\bullet \right)$ is fractional derivative, and ${a}_{\text{p}}$ is the order of fractional derivative which satisfies the condition $0<{a}_{\text{p}}<1$, described as the damping coefficient. The fractional derivatives of a function $f\left(t\right)$ are equal to ${D}_{t}^{a}\left(f\left(t\right)\right)=\frac{{d}^{a}}{d{t}^{a}}f\left(t\right)$. We used Caputo’s definition of the fractional derivative (Podlubny 1999) as follows: ${D}_{t}^{a}\left(f\left(t\right)\right)=\frac{1}{\Gamma \left(1-a\right)}\frac{d}{dt}{\int }_{0}^{t}\frac{f\left({t}^{\prime}\right)}{{\left({t-t}^{\prime}\right)}^{a}}{dt}^{\prime}$ (where $t$ is the independent variable time and $\Gamma \left(1-a\right)$ is the gamma function. If the order of the fractional derivative is $\alpha =0$, then ${D}_{t}^{0}\left(f\left(t\right)\right)=f\left(t\right)$. When $a=1$, the gamma function is $\Gamma \left(1-a\right)\to \infty$. For this case, the fractional derivative is not defined. However, it can be shown that when $a\to 1$, then ${D}_{t}^{a}\left(f\left(t\right)\right)\to \frac{df\left(t\right)}{dt}$.

**Fig. 1:**
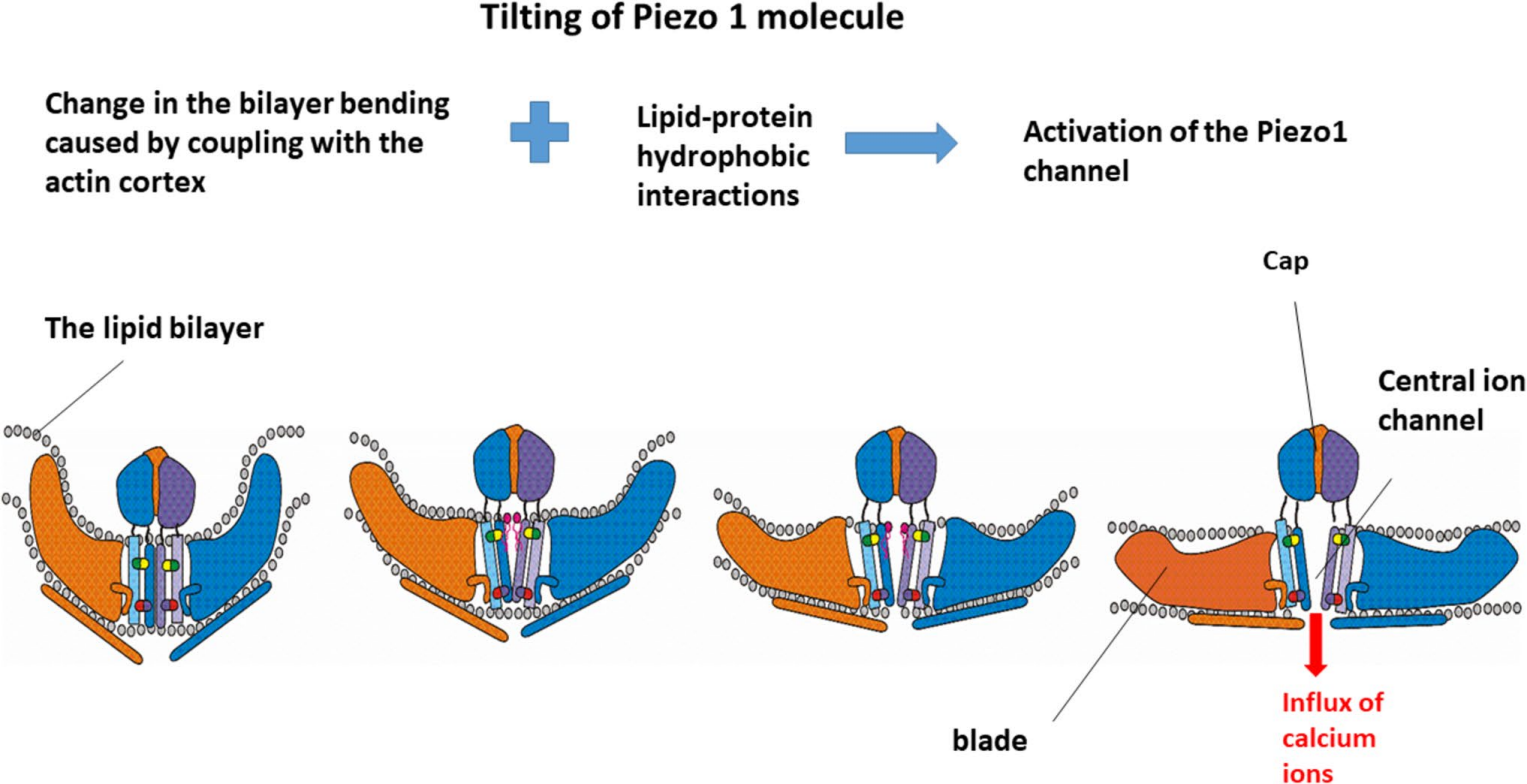
Schematic presentation of the Piezo1 tilting within the bilayer caused by lipid-protein hydrophobic interactions and coupling between the bilayer and cytoskeleton, which impacts the bilayer bending

The diffusion coefficient of Piezo1 depends on the rearrangement of the actin cytoskeleton and is equal to $0.0075\frac{\mu m^2}s$, while the average distance between two Piezo1 molecules in the erythrocyte membrane is 540 ± 37 nm (Vaisey et al. 2022). The distribution of Piezo1 is sensitive to the membrane bending and depends on the membrane curvature (Vaisey et al. 2022; Mulhall et al. 2023). Perozo et al. (2002) examined two mechanisms responsible for the activation of the bacterial mechanosensitive MscL channels. Both mechanisms also apply to other mechanosensitive channels including Piezo1 which like MscL is inherently mechanosensitive (Syeda et al. 2016): (i) the energetic costs associated with mismatches between the protein and the lipid bilayer and (ii) the geometric implications of the intrinsic curvature of the bilayer. The deformation of the lipid bilayer is influenced by several factors: (i) hydrophobic interactions between Piezo1 proteins and lipid molecules, (ii) the coupling between the lipid bilayer and the underlying cytoskeleton, and (iii) the viscoelastic properties of both the cytoskeleton and the bilayer. Furthermore, Vaisey et al. (2022) reported an increase in cholesterol concentration in areas where the bilayer is bending. This increase in cholesterol level surrounding Piezo1 can alter the mobility of Piezo1’s blades and their tilting angle. The tilting of Piezo1 molecules is shown schematically in Fig. 1.

The tilting of the channel protein is affected by both long-range interactions associated with lipid bilayer curvature and short-range lipid compression within its surrounding environment (Killian 1998; Kaiser et al. 2011; Argudo et al., 2016). The angle of tilt is contingent upon cellular tractions, as well as the intensity and direction of externally applied mechanical forces. Given that the interactions between Piezo1 molecules and cholesterol play a crucial role in modulating the conformational changes and activity of Piezo1 (Ridone et al. 2019a, b), it is essential to examine the key elements of these interactions.

## Cholesterol-induced changes in activity of Piezo1 channels

The interaction between cholesterol and Piezo1 molecules occurs via direct, ligand-like, and indirect mechanisms (Zakany et al. 2020). The direct mechanism is related to cholesterol-mediated cross-link of several regions of Piezo1, including the apex of the anchor domain (Buyan et al. 2020). Buyan et al. (2020) pointed out that Piezo1 molecules also form specific, long-lasting interactions with other lipids in the bilayer. Besides direct interactions, cholesterol can affect Piezo1 activity through alterations in the viscoelasticity of the lipid bilayer. Movement of Piezo1’s blades through the bilayer induces generation of compressive mechanical stress, which leads to repulsion among lipids capable of resisting further conformational changes of the channel protein. Leonard et al. (2017) indicated that domains enriched with cholesterol exhibit increased stiffness and are concentrated in areas of high curvature resulting from cellular deformation.

**Fig. 2:**
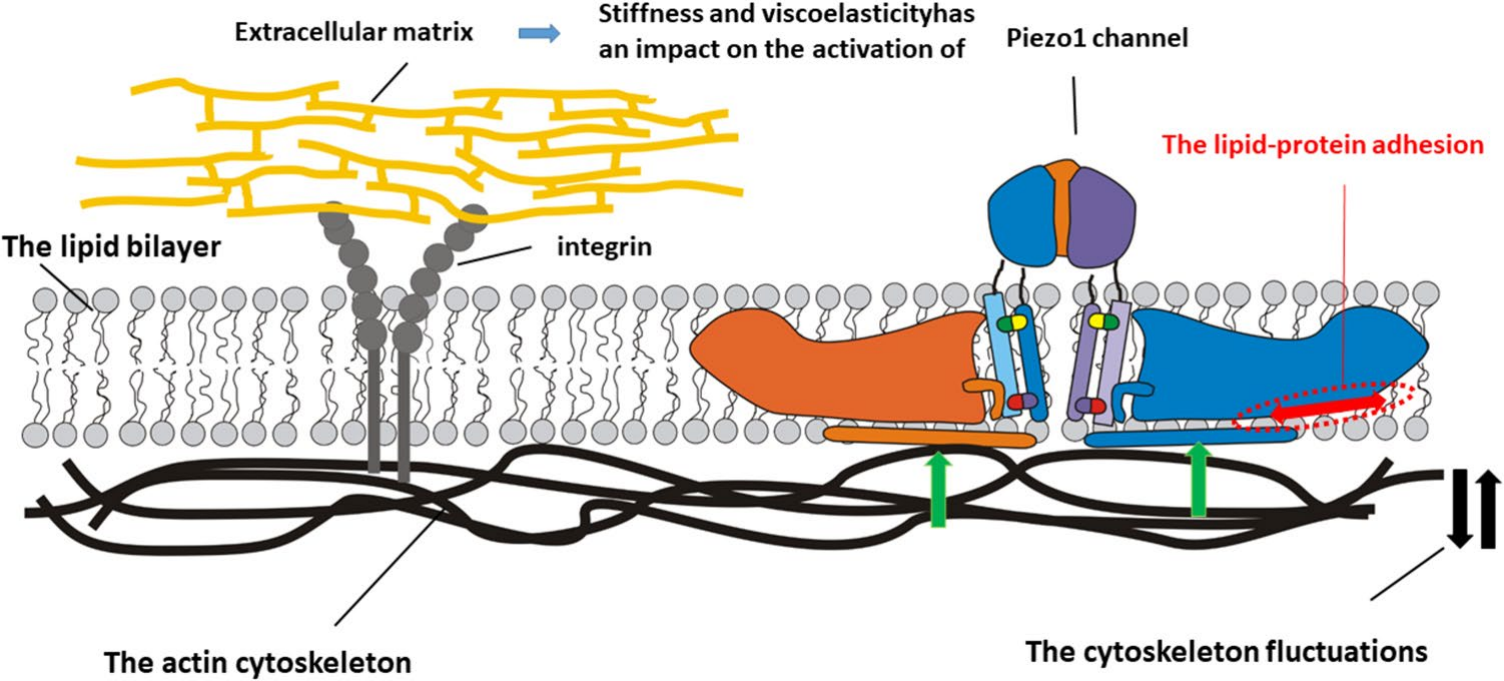
Schematic presentation of two mechanisms that impact the depth of Piezo1 molecules in the bilayer, i.e. the lipid-protein adhesion and the cytoskeleton fluctuations caused by cell tractions and application of external force. Green arrows indicate the orientation of mechanical force, while the red arrow signifies the direction of adhesion force. Additionally, black arrows illustrate the variations occurring within the cytoskeleton

Chong et al. (2021) conducted simulations of the interactions between Piezo1 and cholesterol, revealing that the depth of Piezo1’s footprint decreases in a non-linear manner as cholesterol concentration increases. This observation is consistent with the understanding that increased cholesterol level leads to the stiffening of the lipid bilayer. Notably, this effect is particularly significant at cholesterol concentrations below 10% and above 30%. However, the footprint depth retains an almost constant value for the range of cholesterol concentration from 10 to 30% indicating other mechanisms capable of stabilizing the footprint depth against the bilayer stiffening. This mechanism can be related to the direct interaction between cholesterol and Piezo1. The concentration of cholesterol less than 10% is not enough to produce this stabilizing effect. Two mechanisms, which have an opposite impact to Piezo1’s footprint depth, can be expressed in the form of a force balance as shown in Fig. 2.

Two forces should be accounted for. The mechanical force expressed as ${\overrightarrow{F}}_{m}\left(r,t\right)=\nabla \bullet {\widetilde{\sigma }}_{c}\left(r,t\right)$ is the volumetric driving force of the actin cytoskeleton, which pushes the Piezo1 molecule up, while the lipid-protein adhesion force expressed as ${\overrightarrow{F}}_{\text{a}}\left(r,t\right)={\overrightarrow{\nabla }}_{s}{e}_{\text{a}}$ resists movement of the protein (where $r$ is the space coordinate in surrounding of Piezo1 molecule, ${\widetilde{\sigma }}_{c}\left(r,t\right)$ is the mechanical stress generated within the cytoskeleton in surrounding of Piezo1 molecule, ${e}_{\text{a}}\left(r,t\right)$ is the energy of adhesion between a Piezo1 molecule and surrounding lipids along the biointerface equal to ${e}_{\text{a}}\left(r,t\right)={\rho }_{B}\left(r,t\right)\frac{1}{2}{kx}^{2}$, ${\rho }_{B}$, is the surface number density of the established protein-lipid bonds, $k$ is the spring constant, $x$ is the average length of the protein-lipid bond, and ${\overrightarrow{\nabla }}_{S}$ is the surface gradient). The stress along the cytoskeleton ${\widetilde{\sigma }}_{c}\left(r,t\right)$ accounts for the shear and normal components and depends on the strain generated within the cytoskeleton ${\widetilde{\varepsilon }}_{c}\left(r,t\right)$, which consists of the shear and volumetric components

${\widetilde{\varepsilon }}_{cS}\left(r,t\right)=\frac{1}{2}\left({{\overrightarrow{\nabla }}_{{\overrightarrow{u}}_{c}}+{\overrightarrow{\nabla }}_{{\overrightarrow{u}}_{c}}}^{T}\right)$ and ${\widetilde{\varepsilon }}_{cV}\left(r,t\right)=\left(\overrightarrow{\nabla }\bullet {\overrightarrow{u}}_{c}\right)\widetilde{I}$, respectively, while ${\overrightarrow{u}}_{c}$ is the cytoskeleton displacement field and $\widetilde{I}$ is the unit tensor. An increase in the stiffness of the cytoskeleton under the same strain conditions results in an increase in the stress ${\widetilde{\sigma }}_{c}\left(r,t\right)$. The displacement field of the cytoskeleton ${\overrightarrow{u}}_{c}\left(r,t\right)$ depends on the interplay of (i) the physical properties of the extracellular matrix ECM such as viscoelasticity and stiffness and (ii) the bending of the bilayer surrounding the Piezo1 molecule. This inter-dependence takes place primarily via focal adhesions (FAs), which serve as mechanical transporters between the cell and ECM (Lo 2006). Consequently, the displacement field of ECM caused by single-cell tractions ${\overrightarrow{\text{u}}}_{\text{ECM}}$, which occurs on a time scale of minutes, can be expressed as $\frac{d\Vert {\overrightarrow{u}}_{ECM}\Vert }{dt}=\frac{1}{{\xi }_{ECM}}\frac{{\delta F}_{ECM}}{\delta \Vert {\overrightarrow{u}}_{ECM}\Vert }+\frac{d\Vert {\overrightarrow{u}}_{c}\Vert }{dt}$ (where $\Vert {\overrightarrow{u}}_{ECM}\Vert$ and $\Vert {\overrightarrow{u}}_{c}\Vert$ are the displacement fields ${\overrightarrow{u}}_{ECM}\left(r,t\right)$ and ${\overrightarrow{u}}_{c}\left(r,t\right)$, respectively, ${\xi }_{ECM}$ is the friction coefficient of the matrix protein filaments such as collagen causing their movement induced by cell tractions, and ${\text{F}}_{\text{ECM}}$ is the storage energy within ECM caused by strain generated by cell tractions). Consequently, the membrane fluctuations are influenced by cell tractions depending on the stiffness and viscoelasticity of the ECM.

The cytoskeleton exhibits viscoelastic properties, implying that alterations in its structure due to membrane fluctuations associated with the membrane-ECM coupling during cell tractions result in both energy storage and energy dissipation. The energy storage process contributes to an increase in the stiffness of the cytoskeleton, whereas the dissipation of energy, which occurs through the partial disintegration of the cytoskeletal structure, results in a reduction of its stiffness. The cytoskeleton stiffness depends primarily on the concentration of actin filaments ${n}_{\text{a}}$, their length, and their mobility. Furthermore, the mobility of these filaments is contingent upon both the concentration and the specific types of actin-binding proteins (ABPs) present. The interplay between energy storage and dissipation depends on the applied strain and will be discussed in the context of the constitutive model for the cytoskeleton ${\widetilde{\sigma }}_{c}\left(r,t\right)={\widetilde{\sigma }}_{c}\left({\widetilde{\varepsilon }}_{c}\right)$ in the ‘The bilayer-cytoskeleton coupling on a macroscopic level’ section. The force balance for Piezo1 molecule can be expressed as:

2 $${\xi }_{p}\frac{1}{{V}_{\text{Piezo}1}\left(t\right)}\frac{d{d}_{\text{p}}\left(r,t\right)}{dt}={\overrightarrow{F}}_{\text{a}}\left(r,t\right)\bullet \overrightarrow{t}-{\overrightarrow{F}}_{\text{m}}\left(r,t\right)\bullet \overrightarrow{n}$$

where ${\xi }_{\text{p}}$ is the friction coefficient for movement of Piezo1 molecule through the lipid bilayer, ${V}_{\text{Piezo}1}$ is the average volume of Piezo1 molecule, $\overrightarrow{t}$ is the unit tangential vector, and $\overrightarrow{n}$ is the unit normal vector to the protein-lipid interface. *d*_p_ is Piezo1’s footprint depth, whose equilibrium value ${d}_{\text{p}}\left(r,{t}_{\text{eq}}\right)$, established when $t\to {t}_{\text{eq}}$, depends on the lipid content in the surroundings of the Piezo1 molecule (where ${t}_{\text{eq}}$ is the equilibration time). To fully understand induced changes in the activity of Piezo1 channels, it is necessary to discuss the bilayer-cytoskeleton coupling influenced by the physical properties of the ECM.

## The phenomenological description of cell membrane: the cytoskeleton-bilayer coupling

The cell membrane consists of two main inter-connected layers: the actin cytoskeleton and lipid bilayer. The actin cytoskeleton is intricately linked to the extracellular matrix (ECM) through focal adhesion complexes. These protein complexes play a crucial role in facilitating this connection, thereby influencing the rearrangement of the cytoskeleton and the structural modifications of the matrix caused by the transmission of mechanical forces during cell migration (Ciobanasu et al. 2011). To gain a comprehensive understanding of the mechanosensitivity of Piezo1 channels, which are vital for cellular responses under cyclic stress, it is imperative to examine the interactions between the ECM, the cytoskeleton, and the lipid bilayer (Gaub and Müller 2017). Mechanical properties, such as the stiffness and viscoelasticity of the ECM, significantly affect the strength of focal adhesions, which in turn impacts the reorganization of the cytoskeleton (Lo 2006). Additionally, the deformation of the lipid bilayer surrounding Piezo1 molecules is also contingent upon the interplay among these three components. Given the inter-dependence of the displacements ${\overrightarrow{u}}_{\text{ECM}}$ and ${\overrightarrow{u}}_{\text{c}}$, it follows that the associated strains of the cortex ${\widetilde{\varepsilon }}_{\text{c}}$ and the ECM ${\widetilde{\varepsilon }}_{\text{ECM}}$ are likewise inter-dependent. This mutual relationship among the strains subsequently leads to a corresponding inter-dependence of the stresses generated within the cortex ${\widetilde{\sigma }}_{\text{C}}$ and the ECM ${\widetilde{\sigma }}_{\text{ECM}}$.

The actin cytoskeleton is composed of rigid filaments generated by actin and various actin-binding proteins (ABPs) (Mofrad et al. 2006; Dalhaimer et al. 2007). To date, the specific roles of different ABPs in influencing the mechanical characteristics of the cytoskeleton across diverse cell types have not been thoroughly investigated. ABPs can be distinguished by two main factors: (1) their binding affinity to actin, which is dictated by the unique binding domains they contain, and (2) the configuration, quantity, and organization of their rod-like domains (Wagner et al. 2006). The viscoelastic behaviour of the cytoskeleton is predominantly shaped by several factors, including (1) the flexibility and length of ABPs, (2) the length of the rigid actin filaments, (3) the concentration of these filaments, (4) the mesh size of the actin network, (5) the extent of network rearrangement and uniformity, and (6) the thickness of the cytoskeleton. The cytoskeleton is characterized as an inhomogeneous protein network that comprises small, homogeneous micro-domains (Elson et al. 2010).

Constitutive lipids fail to attain optimal mixing, leading to the formation of either gel and fluid phases or two distinct fluid phases, influenced by external factors such as temperature and tonicity, which refers to the ionic strength of the surrounding solution (Almeida 2009). As a result, the lipid composition within the bilayer exhibits heterogeneity. Furthermore, lipids are arranged into cholesterol-enriched micro-domains known as ‘rafts’, which typically range from 10 to 200 nm in size (Mikhalyov and Samsonov 2011). The viscoelastic characteristics of bilayers in epithelial and cancerous cells display notable distinctions attributed to differences in lipid metabolism. Lipidomic analyses of the highly invasive MDA-MB-231 cell line have indicated an increase in phosphatidylserine (PS), phosphatidylglycerol, and phosphatidic acid, coupled with a decrease in sphingomyelin (SM) when contrasted with the less invasive MCF7 cell line (Wang et al. 2016). In contrast, research conducted by Vidavsky et al. (2019) on the MCF10A cell line series found that invasive cells exhibited elevated levels of sphingomyelin (SM), while phosphatidylethanolamine (PE) and phosphatidylcholine (PC) were less abundant in these cells compared to precancerous cultures. Additionally, Ding et al. (2019) reported an increase in cholesterol concentrations within the membranes of cancerous cells.

The membrane structural changes such as (i) rearrangement of cytoskeleton and (ii) the bending of the bilayer are significantly affected by the viscoelastic properties and stiffness of the ECM. These physical characteristics of the ECM are contingent upon the flexibility and concentration of protein filaments, as well as the density of the inter-filament connections. Collagen networks, which are prevalent ECM components in living organisms, exhibit behaviour characteristic of viscoelastic solids (Pajic-Lijakovic et al. 2022). Cells engaged in migration can actively induce structural changes in ECMs of this type. During their movement, cells exert mechanical forces on the ECM that range from approximately 10 to 100 nN (Emon et al. 2021). This force magnitude significantly exceeds the threshold required to disrupt electrostatic and hydrophobic interactions within collagen I networks, which is estimated to be around 20 pN (Nam et al. 2016). A force of just a few nN is sufficient to elongate collagen filaments by up to 20% (Gautieri et al. 2012). The resultant volumetric reorganization of the collagen I matrix leads to an increase in matrix stiffness (Pajic-Lijakovic et al. 2022). Strain-induced stiffening is a hallmark of collagen networks. The out-of-plane compression of the ECM resulting from cellular tractions leads to the in-plane compression of the ECM and consequently of the cytoskeleton surrounding the Piezo1 molecules. This is one of the primary physical factors that activate Piezo1 channels. This effect is particularly notable in collagen IV networks for pulling forces of a few tens of nN, which can simulate cell tractions (Gaub and Müller 2017).

The relationship between cytoskeletal stiffness and the activity of Piezo1 channels is significantly mediated by cell traction forces. Since this stiffness is influenced by both the density and mobility of actin filaments, it is essential to analyze how the coupling of the cortex with the bilayer affects the density and mobility of actin.

### The bilayer coupling with the actin cytoskeleton on a mesoscopic level

**Fig. 3:**
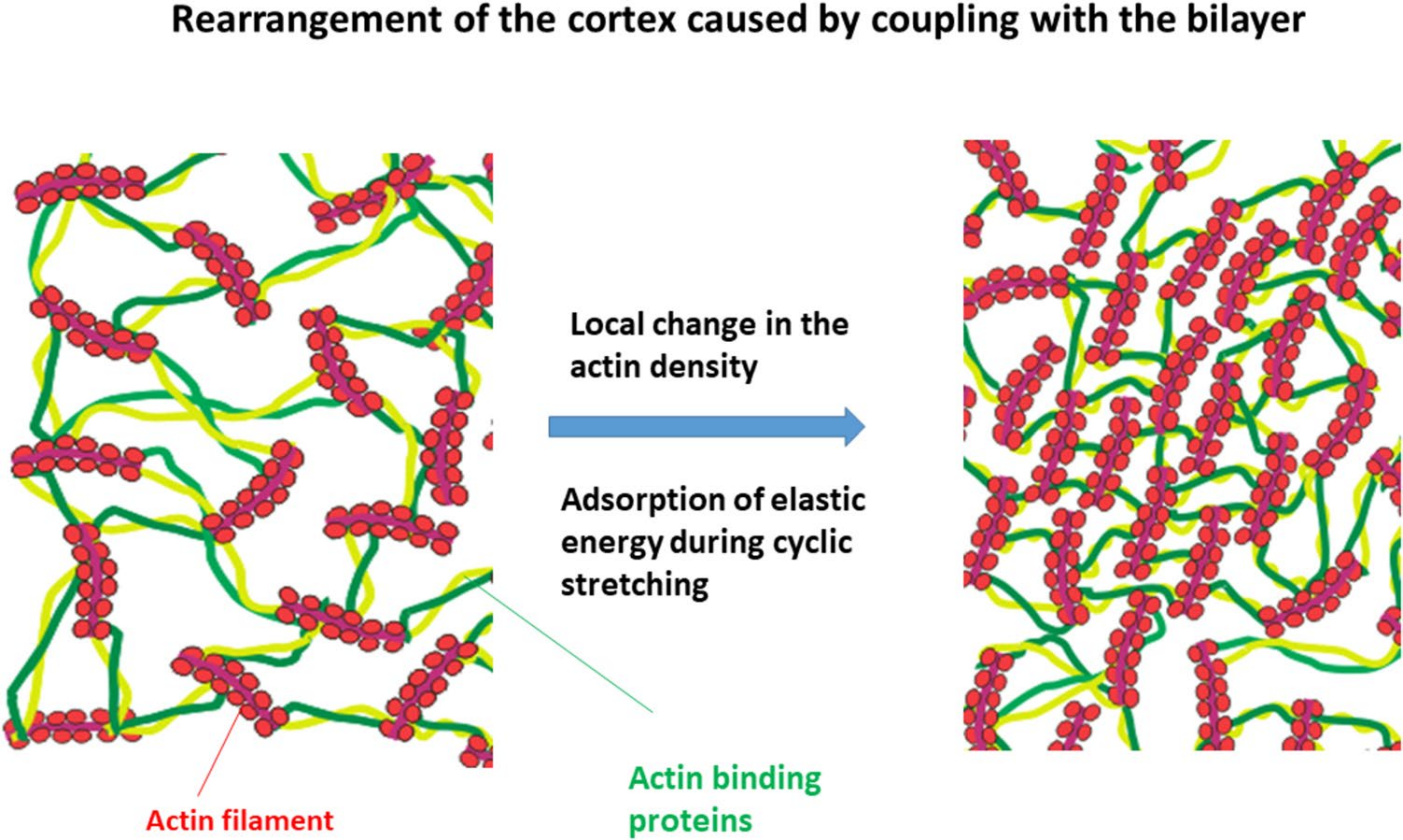
Schematic presentation of change of the number density of actin filaments caused by fluctuations of the membrane during cell tractions and externally applied force. Induced accumulation of elastic energy reduces mobility of actin filaments resulting in the cytoskeleton stiffening

The bilayer coupling with the actin cytoskeleton influences the bilayer bending in the surroundings of Piezo1 molecules. The bilayer bending can be expressed by modified Helfrich type bending free energy functional at a mesoscopic level (Kabaso et al. 2011; Pajic-Lijakovic and Milivojevic 2015) as:

3 $$E\left({n}_{\text{Ra}}\right)=\frac{1}{2}\kappa w\int {\left(\frac{{\partial }^{2}r}{\partial {s}^{2}}-\overline{H}{n }_{\text{Ra}}\right)}^{2}{d}^{2}s$$

where $r$ is the local coordinate; $s$ is the coordinate along the contour; $w$ is the bilayer width; $\kappa$ is the bending modulus, which depends on the hydrodynamic radius of Piezo1 molecule ${r}_{\text{Hp}}$, i.e. $\kappa =\kappa \left({r}_{\text{Hp}}\right)$; $\overline{H }$ is the curvature of the bilayer which depends on the density and mobility of actin filaments; and ${n}_{\text{Ra}}\left(r,s,t\right)$ is the dimensionless density of actin filaments within the cytoskeleton relative to the maximum density, i.e. ${n}_{\text{Ra}}\left(r,s,t\right)=\frac{{n}_{\text{a}}}{{n}_{\text{a max}}}$. The movement of actin filaments caused by cell tractions and externally induced mechanical force can be expressed as (Kabaso et al. 2011):

4 $$\frac{1}{\dot{s }}\frac{\partial \left(\dot{s }{n}_{\text{a}}\right)}{\partial t}=\frac{{D}_{a}}{\dot{s }}{\nabla }_{s}^{2}\left(\dot{s }{n}_{\text{a}}\right)+\frac{1}{\dot{s }}\frac{{\mu }_{\text{a}}}{{n}_{\text{a max}}}{\nabla }_{\text{s}}\left(\dot{s }{n}_{\text{a}}{\nabla }_{\text{s}}\left(\frac{1}{\dot{s }}\frac{\delta E}{\delta {n}_{\text{a}}}\right)\right)$$

where ${D}_{\text{a}}=\frac{{k}_{B}T}{{\xi }_{\text{a}}}$ is the diffusion coefficient of actin, ${k}_{\text{B}}$ is Boltzmann constant, $T$ is temperature, $\dot{s}=\frac{ds}{dt}$, ${\nabla }_{\text{s}}$ is the derivative along the contour, and ${\mu }_{\text{a}}$ is the mobility of actin filaments equal to ${\mu }_{\text{a}}={{\xi }_{\text{a}}}^{-1}$. The mobility of actin filaments is influenced by the concentration of actin-binding proteins (ABPs) and is observed to decrease as elastic energy accumulates within the cytoskeletal structure. The energy stored in the cytoskeleton is contingent upon the fluctuations of the membrane, which are in turn affected by the stiffness of the extracellular matrix (ECM) and the robustness of focal adhesions (FAs). It means that intensive accumulation of elastic energy, i.e. the strain stiffening, reduces the mobility of actin and causes the stiffening of the cytoskeleton, which have a feedback impact on the bilayer bending. Local change of the number density of actin filaments caused by fluctuations of the membrane is shown in Fig. 3.

For a better understanding of this cause-consequence cycle, it is necessary to discuss the viscoelasticity of the cell membrane.

### The bilayer-cytoskeleton coupling on a macroscopic level

The estimation of an appropriate way for the coupling between the bilayer and the cytoskeleton is essential for accurately characterizing the viscoelastic properties of the cell membrane. Lamellar structure of the membrane indicates parallel coupling between the lipid bilayer and cytoskeleton expressed as (Pajic-Lijakovic and Milivojevic 2015):

5 $${\widetilde{\sigma }}_{\text{m}}\left(r,t\right)={\widetilde{\sigma }}_{\text{C}}\left(r,t\right)+{\widetilde{\sigma }}_{\text{L}}\left(r,t\right)$$

where ${\widetilde{\sigma }}_{\text{m}}$ is the mechanical stress within the membrane, ${\widetilde{\sigma }}_{\text{C}}$ is the cytoskeleton contribution to the membrane stress, and ${\widetilde{\sigma }}_{\text{L}}$ is the bilayer contribution to the membrane stress. The corresponding strains, for the proposed way of coupling, satisfy the condition that:

6 $${\widetilde{\varepsilon }}_{\text{m}}\left(r,t\right)={\widetilde{\varepsilon }}_{\text{C}}\left(r,t\right)={\widetilde{\varepsilon }}_{\text{L}}\left(r,t\right)$$

where ${\widetilde{\varepsilon }}_{\text{m}}$ is the strain within the membrane, ${\widetilde{\varepsilon }}_{\text{C}}$ is the strain within the cytoskeleton, and ${\widetilde{\varepsilon }}_{\text{L}}$ is the strain within the bilayer. The strain ${\widetilde{\varepsilon }}_{\text{m}}$ consists of the shear component equal to ${\widetilde{\varepsilon }}_{\text{mS}}\left(r,t\right)=\frac{1}{2}\left(\overrightarrow{\nabla }{\overrightarrow{u}}_{\text{c}}+{\overrightarrow{\nabla }{\overrightarrow{u}}_{c}}^{T}\right)$ and volumetric component equal to ${\widetilde{\varepsilon }}_{\text{mV}}\left(r,t\right)=\overrightarrow{(\nabla }\bullet {\overrightarrow{u}}_{\text{c}})\widetilde{I}$, and $\widetilde{I}$ is the unit tensor. The membrane strain components depend on the corresponding strain components of the ECM. It is in accordance with the fact that the displacement fields ${\overrightarrow{u}}_{\text{c}}$ and ${\overrightarrow{u}}_{\text{ECM}}$ are inter-related. The corresponding constitutive models for the actin cytoskeleton, as well as for the membrane as a whole, can be formulated based on the main characteristics of the membrane viscoelasticity for epithelial and cancer cells discussed in the literature.

## Viscoelasticity of the membrane: the main characteristics

The viscoelasticity of the membrane has been measured by active and passive micro rheological techniques and discussed in the context of storage and loss moduli vs. frequency, i.e. the frequency sweep. The storage modulus represents the measure of energy storage, whereas the loss modulus is the measure of energy dissipation during structural changes of the lipid bilayer and actin cytoskeleton. Frequency sweep experiments usually cover the frequency range from 1 to 100 Hz, while the cytoskeleton stress relaxation time under constant strain conditions corresponds to a few minutes (Xu et al. 2000). As a result, the stress induced by the application of oscillatory strain does not have sufficient time to relax. Experimental data for the storage and loss moduli of the cell membranes of breast epithelial MCF10A cells and cancer MDA-MB-231 cells as a function of angular velocity from Rother et al. (2014) and Hang et al. (2022) show three important characteristics:

(i) The ratio between storage and loss moduli of the membrane $\frac{{G}_{m}{\prime}\left(r,\omega \right)}{{G}_{m}^{{\prime}{\prime}}\left(r,\omega \right)}$ is (1) $\frac{{G}_{m}{\prime}\left(\omega \right)}{{G}_{m}^{{\prime}{\prime}}\left(\omega \right)}>1$, indicating solid-like behaviour for frequencies lower than ${f}_{\text{c}}$ and $\frac{{G}_{m}{\prime}\left(r,\omega \right)}{{G}_{m}^{{\prime}{\prime}}\left(r,\omega \right)}<1$ (regime 1), and (2) $\frac{{G}_{m}{\prime}\left(r,\omega \right)}{{G}_{m}^{{\prime}{\prime}}\left(r,\omega \right)}<1$, which signifies liquid-like behaviour for frequencies higher than ${f}_{\text{c}}$ (regime 2) (where ${G}_{m}{\prime}\left(r,\omega \right)$ is the storage modulus, ${G}_{m}^{{\prime}{\prime}}\left(r,\omega \right)$ is loss modulus, and $\omega$ is the angular velocity equal to $\omega =2\pi f$). Characteristic frequency ${f}_{\text{c}}$ for MCF10A cells was ${f}_{\text{c}}\sim 60 \text{Hz}$, while for cancer MDA-MB-231 cells, this frequency is almost three times lower (Rother et al. 2014).
(ii) The anomalous nature of energy dissipation and energy storage observed in regime 1 can be described by fractional derivatives (Rother et al. 2014; Pajic-Lijakovic and Milivojevic 2015; Hang et al. 2022). This anomalous energy dissipation arises from two primary factors: (1) sub-diffusion of lipids within the bilayer and hop diffusion between lipid rafts and (2) conformational changes of semi-flexible proteins within the cytoskeleton caused by the bilayer-cytoskeleton coupling (Fujiwara et al., 2002; 2016). Spatial distribution of lipid molecules and their conformations depend on the bilayer bending state (Leonard et al. 2017). For higher frequencies, i.e. $f>{f}_{\text{c}}$ in regime 2, the energy dissipation rather represents a consequence of the disruption of weak inter-chain bonds that constitute the cytoskeleton.
(iii) The storage modulus exhibits an increase with frequency in regime 1, whereas in regime 2, it approaches a nearly constant value. The scaling exponent in regime 1 is less than 1 (Hang et al. 2022).
(iv) The loss modulus exhibits an increase in both regimes. The scaling exponent for regime 1 closely resembles that of the storage modulus. In contrast, the rise in the loss modulus in regime 2 is more pronounced, characterized by a scaling exponent of one (Rother et al. 2014; Hang et al. 2022).
(v) The storage modulus of MCF10A cells is markedly greater than that of MDA-MB-231 cells, whereas the loss modulus of MDA-MB-231 cells is considerably higher compared to that of MCF10A cells. This result indicates that the rearrangement of the membrane of MDA-MB-231 cells is more dissipative and the mobility of actin filaments within the cytoskeleton of MCF10A cells is reduced.

### Constitutive model for the cell membrane

The rheological behaviour of the bilayer is primarily affected by its fluidity caused by sub-diffusion of lipids, while the bilayer bending has been treated as an elastic deformation (Wu et al. 2015). Consequently, the bilayer behaves viscoelastically (Wu et al. 2015). The viscoelasticity of the bilayer depends on (i) the lipid content, (ii) viscoelasticity of the actin cytoskeleton, and (iii) the way of the bilayer-cytoskeleton coupling (Pajic-Lijakovic and Milivojevic 2015; Ridone et al. 2019a, b). In accordance with the fact that sub-diffusion of lipids causes the anomalous dissipation of energy, Pajic-Lijakovic and Milivojevic (2014) proposed the following constitutive model:

7 $${\widetilde{\sigma }}_{\text{L}}\left(r,t\right)={\eta }_{a\text{L}}{D}_{t}^{{a}_{L}} {\widetilde{\varepsilon }}_{\text{m}}$$

where ${\widetilde{\varepsilon }}_{\text{m}}$ is the strain of the bilayer caused by the presence of Piezo1 molecule, ${\eta }_{aL}$ is the effective modulus of the lipid bilayer, ${D}_{t}^{a} \left(\bullet \right)$ is the corresponding fractional derivative, and ${a}_{\text{L}}$ is the order of fractional derivative (Pajic-Lijakovic and Milivojevic 2015). For a fractional derivative higher than zero and lower than one, this equation describes damped dissipative phenomena caused by sub-diffusion of lipids. Fractional derivatives account for the non-linear nature of the membrane viscoelasticity.

The constitutive model for the actin cytoskeleton formulated by Pajic-Lijakovic and Milivojevic (2015) is:

8 $${\widetilde{\sigma }}_{\text{C}}\left(r,t\right)={G}_{\text{sC}}{\widetilde{\varepsilon }}_{\text{m}}+{\eta }_{a\text{C}}{D}_{t}^{{a}_{C}} {\widetilde{\varepsilon }}_{\text{m}}+{\eta }_{C}{\dot{\widetilde{\varepsilon }}}_{\text{m}}$$

where ${G}_{\text{sC}}$ is the elastic shear modulus of the cytoskeleton equal to ${G}_{\text{sC}}=\frac{{k}_{B}T}{\langle {l}_{d}^{3}\rangle }$, $\langle {l}_{d}^{3}\rangle$ is the average cytoskeleton micro-domain volume, ${\alpha }_{\text{C}}$ is the damping coefficient caused by conformational changes of semi-flexible protein filaments within the cytoskeleton, ${\eta }_{a\text{C}}$ is the effective modulus of the cytoskeleton, ${\eta }_{\text{C}}$ is the viscosity of the cytoskeleton, and ${\dot{\widetilde{\varepsilon }}}_{\text{m}}$ is the strain rate equal to ${\dot{\widetilde{\varepsilon }}}_{\text{m}}=\frac{d{\widetilde{\varepsilon }}_{\text{m}}}{dt}$. A higher value of the modulus ${G}_{\text{sC}}$ points to a stiffer cytoskeleton structure. In accordance with the fact that the rearrangement of the lipid bilayer is more dissipative than the rearrangement of the actin cytoskeleton, the following relationship between the damping coefficients of the bilayer and the cytoskeleton can be established: ${\alpha }_{\text{L}}>{\alpha }_{\text{C}}$. Total stress within the membrane can be expressed by introducing Eqs. 7 and 8 into Eq. 5:

9 $${\widetilde{\sigma }}_{\text{m}}\left(r,t\right)={G}_{\text{sC}}{\widetilde{\varepsilon }}_{\text{m}}+{\eta }_{a\text{m}}{D}_{t}^{a} {\widetilde{\varepsilon }}_{\text{m}}+{\eta }_{\text{C}}{\dot{\widetilde{\varepsilon }}}_{\text{m}}$$

where ${\eta }_{a\text{m}}$ is the effective modulus, which accounts anomalous nature of structural changes of both, the bilayer and the actin cytoskeleton. The second term on the right-hand side of Eq. 9 is expressed as ${\eta }_{am}{D}_{t}^{a}{\widetilde{\varepsilon }}_{m}\approx {\eta }_{aC}{D}_{t}^{aC} {\widetilde{\varepsilon }}_{m}+{\eta }_{aL}{D}_{t}^{aL} {\widetilde{\varepsilon }}_{m}$ by introducing resulting effective modulus of the membrane ${\eta }_{a\text{m}}$ accompanied by the damping coefficient $a$. This is the way to reduce the number of model parameters. This constitutive model (Eq. 9) has been used for modelling the viscoelasticity of the membrane of (i) human alveolar A549 cells and (ii) muscle cells based on experimental data from Alcaraz et al. (2003) and Fabry et al. (2001), respectively. The first term on the right-hand side of Eq. 9 includes elastic contribution to mechanical stress caused by reversible structural changes of the cytoskeleton. The second term accounts for the anomalous nature of energy storage and energy dissipation during the membrane structural changes. The third term includes viscous contribution to the mechanical stress caused by irreversible structural changes of the cytoskeleton primarily related to the disruption of weak inter-filament bonds during protein conformations. The fraction of active population of Piezo1 molecules along the membrane $y\left(r,t\right)$ should correlate with the divergence of the mechanical stress, i.e. $y\left(r,t\right)\sim \nabla {\widetilde{\sigma }}_{\text{m}}\left(r,t\right)$. This relationship merits additional exploration.

The model in Eq. 9 can be transformed from the time domain into the frequency domain using the Fourier integral transform. Transforming equation is expressed in the form $F\left[{\widetilde{\sigma }}_{\text{m}}\right]={G}_{m}^{*}\left(r,\omega \right)F\left[{\widetilde{\varepsilon }}_{\text{m}}\right]$ (where $F\left[\bullet \right]$ is the Fourier transform, $\omega$ is the angular velocity, and ${G}_{m}^{*}\left(r,\omega \right)$ is the complex modulus of the bilayer equal to ${G}_{m}^{*}={G}_{m}{\prime}+i{G}_{m}^{{\prime}{\prime}}$ (where ${G}_{m}{\prime}\left(r,\omega \right)$ is the storage modulus, ${G}_{m}^{{\prime}{\prime}}\left(r,\omega \right)$ is the loss modulus, and $i=\sqrt{-1}$ is the imaginary unit)). The storage modulus quantifies elastic behaviour and the loss modulus quantifies viscous behaviour of the membrane. Consequently, a higher storage modulus relative to the loss modulus indicates a stiffer membrane structure. The viscoelasticity of the membrane, quantified by the model parameters ${G}_{\text{sC}}$, ${\eta }_{a\text{m}}$, ${\eta }_{\text{C}}$, and $a$, depends on the membrane-ECM coupling. Corresponding storage and loss moduli for the whole membrane are equal to:

10a $${G}_{m}{\prime}\left(\omega \right)={G}_{\text{sC}}+{\eta }_{a\text{m}}{\omega }^{a}cos\left(\frac{\pi \alpha }{2}\right)$$

10b $${G}_{m}^{{\prime}{\prime}}\left(\omega \right)={\eta }_{a\text{m}}{\omega }^{a}\text{sin}\left(\frac{\pi a}{2}\right)+{\eta }_{C}\omega$$

where $\omega$ is the angular velocity equal to $\omega =2\pi f$. While the first term on the right-hand side of Eq. 10b dominantly influences an increase in loss modulus caused by anomalous nature of energy dissipation in regime 1, the second term is dominant in regime 2. The four model parameters ${G}_{\text{sC}}$, ${\eta }_{a\text{m}}$, ${\eta }_{\text{C}}$, and $a$ were obtained by independent comparative analyses of the two experimental data sets ${G}_{m}{\prime}\left(r,\omega \right)$ vs. $\omega$ and ${G}_{m}^{{\prime}{\prime}}\left(r,\omega \right)$ vs. $\omega$. The calculated values should be fitted with the experimental data by minimizing the squared magnitude of the residuals of the storage and loss moduli in order to determine the optimal values of the model parameters.

In this theoretical analysis, we aim to highlight the key features of the viscoelastic properties of epithelial and cancer cell membranes, specifically focusing on breast epithelial MCF10A cells and MDA-MB-231 cells. This discussion is grounded in experimental findings reported by Rother et al. (2014), which were obtained under consistent conditions. The experimental data and model predictions are presented in Appendix.

## The interplay between the main factors responsible for selective killing of cancer cells under cyclic stretch

**Fig. 4:**
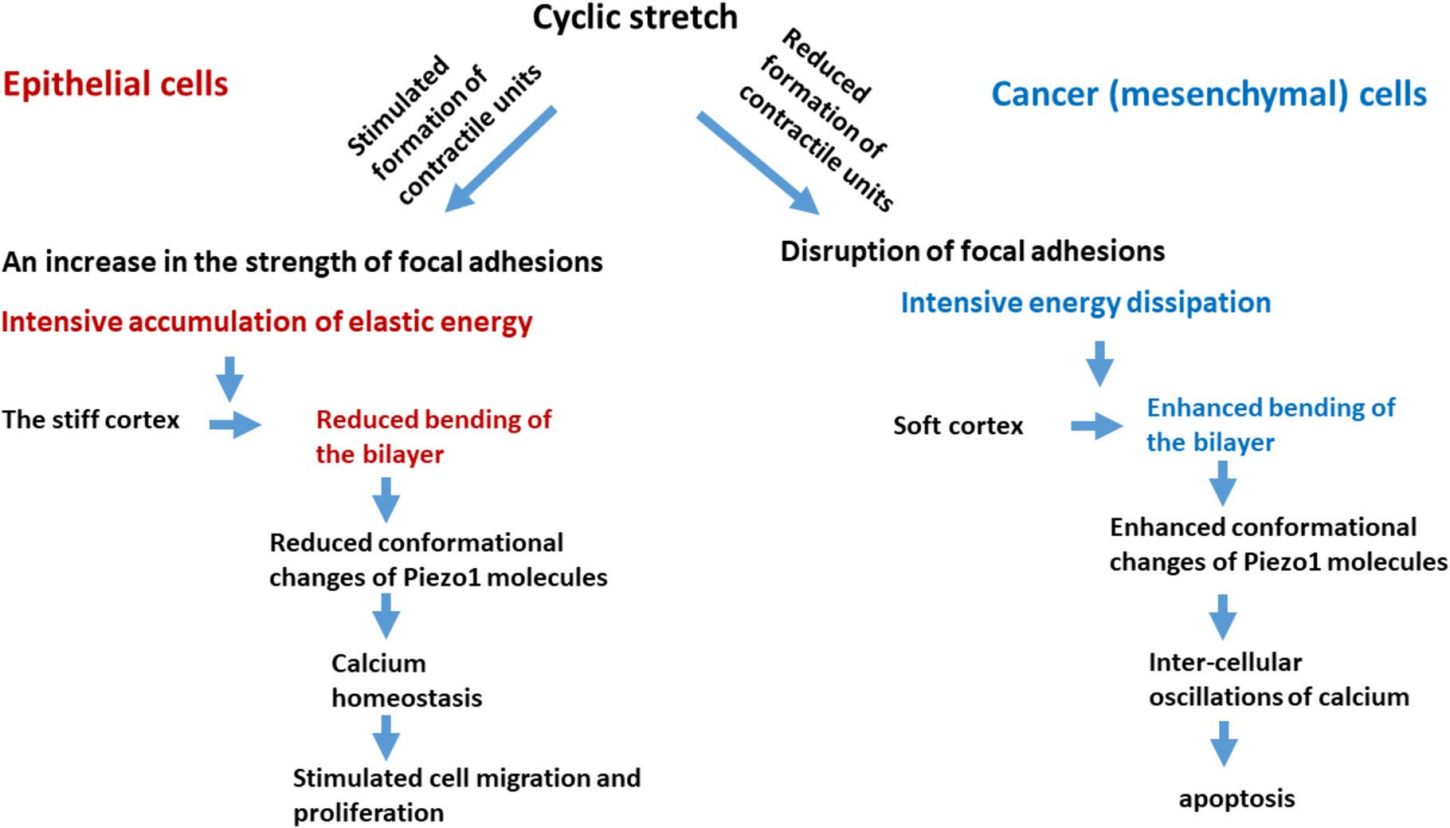
Schematic presentation of the responses of epithelial and cancer cells subjected to cyclic stretch, within the framework of interrelationship between various physical parameters

Mesenchymal-like cancer cells exhibit several distinctive characteristics that set them apart from normal cells, which are essential for comprehending their behaviour under cyclic stretch. These inter-connected features include (i) a reduction in the stiffness of the actin cytoskeleton; (ii) increase in the concentration of cholesterol in the bilayer; (iii) significant energy dissipation during the rearrangement of the cytoskeleton, closely associated with its viscoelastic behaviour; (iv) the formation of a restricted number of contractile units; (v) the development of smaller focal adhesions; (vi) establishment of weak cell–cell adhesion contacts; (vii) pronounced cell tractions, which depend on the stiffness and viscoelasticity of the ECM; and (viii) spontaneous oscillations in intracellular calcium levels. Spontaneous intracellular calcium oscillations are caused by traction-induced perturbation of Piezo1 channels. Cyclic stretch (i) triggers an increase in expression of Piezo1 molecules, (ii) additionally perturbs the influx of calcium through Piezo1 channels, and (iii) induces disruption of FAs depending on the physical properties of the ECM, which represent the main reasons for the apoptosis of cancer cells. The conformational alterations of Piezo1 molecules are influenced by the bending of the lipid bilayer. This bending is contingent upon (i) the lipid composition, the viscoelastic properties of the cellular cytoskeleton, and (iii) physical properties of the ECM. Specifically, an increase in cholesterol content leads to a reduction in bilayer bending, whereas significant energy dissipation during the rearrangement of the cytoskeleton promotes greater bilayer bending. This energy dissipation arises from interactions occurring both between and within semi-flexible filaments. As a result, the adhesive interactions between Piezo1 molecules and lipids fail to provide stability to the Piezo1 molecules. Schematic presentation of the response of epithelial and cancer cells under cyclic stretch in the context of relevant physical factors is shown in Fig. 4.

Interestingly, epithelial cells exhibit a remarkable ability to protect Piezo1 molecules from conformational perturbations induced by mechanical forces, which could otherwise result in an uncontrolled influx of calcium ions. The differential responses observed between epithelial and cancer cells can be attributed to the viscoelastic properties of their membranes. Under identical experimental conditions, the cytoskeleton of epithelial cells is capable of storing greater elastic energy compared to that of cancer cells, which, conversely, demonstrates a higher rate of energy dissipation. This accumulation of elastic energy in epithelial cells leads to a reduction in the mobility of actin filaments, contributing to the stiffening of the cytoskeleton. This stiffening effect, in turn, reduces the bending of the lipid bilayer. A decrease in the bending of the bilayer surrounding Piezo1 molecules is essential for optimizing the functionality of Piezo1 channels.

The following experiments can help to further strengthen the proposed concepts:

(i) Determination of mechanical cell properties of primary cells and cancer cells and comparing their viscoelastic properties can be done by stretching the cells using acoustic force spectroscopy (AFS) as previously reported for HEK293T cells (Romanov et al. 2020). The advantage of this method is that multiple cells can be studied simultaneously enabling rapid measurements of viscoelastic properties of both primary and cancer cells.
(ii) Furthermore, to conduct additional experiments examining the viscoelastic response of the cell membrane during cyclic stretching and determining the residual stress accumulation contributing to the cytoskeleton stiffening, we can employ IsoStretcher, which is an isotropic cell stretch device allowing cyclic stretching of cells (Schürmann et al. 2016). The IsoStretcher method also allows to examine Piezo1 activity measuring Ca2 + fluorescence (Merten et al. 2024). Mechanical properties of the stretched cells after exposure to different cyclic stretch regimes can then be determined using AFS.

## Conclusion

The application of cyclic stretch may provide an innovative therapeutic approach against cancer development. This mechanical stimulus has been shown to selectively induce cell death in mesenchymal-like cancer cells while enhancing the migration and proliferation of epithelial cells. Although the underlying mechanisms have been discussed in terms of cell signalling, gene expression, and biochemical processes, there remains a significant gap in understanding the physical factors that drive cellular responses. This study intends to shed light on the role of physical factors, specifically the viscoelastic characteristics of the actin cytoskeleton and lipid bilayer, and how their coupling influences bilayer bending in response to cyclic stretch in epithelial and cancer cells. The key results are derived from a discussion of cellular responses to cyclic stretch, emphasizing the physical interactions between the bilayer, cytoskeleton, and mechanosensitive Piezo1 channels. These results can be summarized as follows:

- Cyclic stretching promotes the expression of Piezo1 molecules in cancer cells, while it does not affect the expression of Piezo1 molecules in epithelial cells.
- The development of contractile units, which are essential for the stabilization of focal adhesions (FAs), is reduced in cancer cells, while being augmented in epithelial cells subjected to cyclic stretch. This deficiency in contractile units may result in the destabilization of FAs, ultimately leading to the apoptosis of cancer cells. Conversely, cyclic stretch contributes to the increased strength of FAs in epithelial cells, which is pronounced on stiffer ECMs.
- The conformational dynamics of Piezo1 molecules are determined by the lipid composition and the bending of the lipid bilayer. Pronounced bending of the bilayer can perturb the state of Piezo1 channels, resulting in an uncontrollable increase in calcium influx that may perturb intracellular calcium homeostasis in cancer cells. This mechanically induced perturbation is a significant contributor to the apoptosis of cancer cells.
- The bending of the bilayer surrounding Piezo1 molecules is significantly increased in cancer cells, in contrast to epithelial cells. This phenomenon can be attributed to the distinct rheological properties exhibited by the membranes of these two cell types. In epithelial cells, cyclic stretching leads to an accumulation of elastic energy within the actin cytoskeleton, which in turn restricts the mobility of actin filaments. The accumulation of energy within the cytoskeleton is a product of the cytoskeleton coupling with the ECM and depends on its viscoelasticity and stiffness. The resultant mechanical stiffening of the cytoskeleton affects the bilayer-cytoskeleton coupling, thereby reducing the bending of the bilayer in epithelial membranes. Conversely, in cancer cells, the substantial energy dissipation resulting from the disruption of certain inter-filament bonds during cortical rearrangement under cyclic stretch enhances the mobility of actin filaments, consequently increasing the bending of the bilayer.

It is essential to conduct additional experiments to evaluate the rheological response of the membrane during cyclic stretching in the time domain and to measure the residual stress accumulation within the cytoskeleton for each cell type as the main contributor of the cytoskeleton stiffening.

T151 is a type of epithelial cells; Caco-2 and IEC-6 are intestinal epithelial cell types; $\uparrow$ indicates an enhancement in both cell migration and proliferation; $\uparrow$ indicates a decrease in cell migration and proliferation, along with an enhancement of cell apoptosis, and $\sim$ points out that some cell types such as HT29 (Strelez et al. 2021) and T151 (Dow et al. 2024) are less sensitive to cyclic stretching.

*HUVEC* human umbilical vein endothelial cell, *MDCK* Madin-Darby canine kidney epithelial cell, *SKOV3* human ovarian adenocarcinoma, *HT1080* human fibrosarcoma, *HEK293T* transformed human embryonic kidney cell, *MCF10A* human breast epithelial cell, *MDA-MB-231* metastatic breast cancer cell line, *MCF-7* human non-metastatic breast adenocarcinoma cells.

## Figures and Tables

**Fig. 5:**
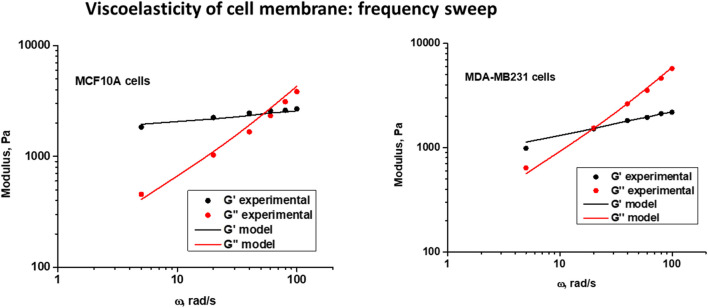
Storage and loss moduli vs. angular velocity for epithelial MCF10A cell and cancer MDA-MB-231 cell, experimental data and model prediction

## Data Availability

Microrheological data in the form of storage and loss moduli vs. angular velocity for MCF10A cells and MDA-MB-231 cells from Rother et al. (2014)Rother et al. (2014) Atomic force microscopy-based microrheology reveals significant differences in the viscoelastic response between malign and benign cell lines. Open Biol. 4: 140046, http://dx.doi.org/10.1098/rsob.140046) were compared with our calculations obtained by using developed model proposed here to: (i) illustrate the usefulness of the model proposed and (ii) to discuss the difference in rheological response of these cell types based on model parameters calculated in this procedure.

## Appendix 1

The characterization of the viscoelastic properties of the membranes in both epithelial and cancer cells by using the proposed constitutive model.

Corresponding experimental data of storage and loss moduli vs. angular velocity for the epithelial and cancer membranes from Rother et al. (2014) were compared with the model predictions using Eqs. 10a and 10b. Corresponding experimental data and model predictions are shown in Fig. 5.

The model correlated with the experimental data for (i) MCF1-A cells with the standard deviation SD = ± 0.0127 for the storage modulus and SD = ± 0.0349 for loss modulus and (ii) MDA-MB-231 cells with the standard deviation SD = ± 0.0271 for the storage modulus and SD = ± 0.0412 for loss modulus.

The corresponding values of the model parameters are shown in Table 2.

**Table 2:** Model parameters from constitutive model Eqs. 10a and 10b for breast epithelial MCF10A cell and cancer MDA-MB-231 cell

Model parameters	MCF10A cell	MDA-MB-231 cell
${G}_{\text{sC}} (\text{Pa})$	$1500\pm 50$	$700\pm 50$
${\eta }_{a\text{m}} ({\text{Pas}}^{a})$	$185\pm 10$	$190\pm 10$
${\eta }_{\text{C}} (\text{Pas})$	$6\pm 1$	$8\pm 1$
$a$(-)	$0.29\pm 0.01$	$0.34\pm 0.01$

The elastic shear modulus ${G}_{\text{sC}}$ of the cortical region is nearly twice as high in epithelial MCF10A cells compared to cancerous MDA-MB-231 cells. Tijore et al. (2021) further emphasized that the cytoskeleton of cancer cells exhibits a significantly reduced stiffness relative to that of epithelial cells. The modifications in membrane structure reveal enhanced dissipative properties in cancer cells when contrasted with healthy epithelial cells, even under identical rates of strain variation. This increased energy dissipation in cancer cells is reflected in increased values of the cytoskeleton viscosity ${\eta }_{\text{C}}$ and the damping coefficient *α*. The viscosity of the cross-linked actin extracted from cells is approximately 15 Pas (Murrell et al. 2011). Additionally, cancer cells display alterations in membrane lipid composition when compared to epithelial cells, which can be attributed to dysregulated lipid metabolism (Maja et al. 2022). These changes are indicated by a higher damping coefficient *α*, while the effective modulus ${\eta }_{a\text{m}}$ remains nearly constant across both cell types.

As a result, MCF10A cells accumulate a considerably greater quantity of elastic energy within their cell membranes when compared to MDA-MB-231 cells. This accumulation leads to the stiffening of the cytoskeleton, which is associated with a decrease in the mobility of actin filaments. This change exerts a feedback effect on the bending of the bilayer and the mobility of lipids, which in turn affects the activity of the Piezo1 channels.
